# *Escherichia coli *phylogenetic group determination and its application in the identification of the major animal source of fecal contamination

**DOI:** 10.1186/1471-2180-10-161

**Published:** 2010-06-01

**Authors:** Camila Carlos, Mathias M Pires, Nancy C Stoppe, Elayse M Hachich, Maria IZ Sato, Tânia AT Gomes, Luiz A Amaral, Laura MM Ottoboni

**Affiliations:** 1 Centro de Biologia Molecular e Engenharia Genética, Universidade Estadual de Campinas - UNICAMP, C. P. 6010, 13083-875 Campinas, S. P., Brasil; 2 Programa de Pós Graduação em Ecologia, Instituto de Biologia, Universidade Estadual de Campinas -- UNICAMP, 13083-970 Campinas, S. P., Brasil; 3 Departamento de Análises Ambientais, Companhia Ambiental do Estado de São Paulo - CETESB, 05459-900 São Paulo, S. P., Brasil; 4 Departamento de Microbiologia, Imunologia e Parasitologia, Universidade Federal de São Paulo -- UNIFESP, 04023-062 São Paulo, S. P., Brasil; 5 Faculdade de Ciências Agrárias e Veterinárias, Universidade Estadual Paulista -- UNESP, 14884-900 Jaboticabal, S. P., Brasil

## Abstract

**Background:**

*Escherichia coli *strains are commonly found in the gut microflora of warm-blooded animals. These strains can be assigned to one of the four main phylogenetic groups, A, B1, B2 and D, which can be divided into seven subgroups (A_0_, A_1_, B1, B2_2_, B2_3_, D_1 _and D_2_), according to the combination of the three genetic markers *chuA*, *yjaA *and DNA fragment TspE4.C2. Distinct studies have demonstrated that these phylo-groups differ in the presence of virulence factors, ecological niches and life-history. Therefore, the aim of this work was to analyze the distribution of these *E. coli *phylo-groups in 94 human strains, 13 chicken strains, 50 cow strains, 16 goat strains, 39 pig strains and 29 sheep strains and to verify the potential of this analysis to investigate the source of fecal contamination.

**Results:**

The results indicated that the distribution of phylogenetic groups, subgroups and genetic markers is non-random in the hosts analyzed. Strains from group B1 were present in all hosts analyzed but were more prevalent in cow, goat and sheep samples. Subgroup B2_3 _was only found in human samples. The diversity and the similarity indexes have indicated a similarity between the *E. coli *population structure of human and pig samples and among cow, goat and sheep samples. Correspondence analysis using contingence tables of subgroups, groups and genetic markers frequencies allowed the visualization of the differences among animal samples and the identification of the animal source of an external validation set. The classifier tools Binary logistic regression and Partial least square -- discriminant analysis, using the genetic markers profile of the strains, differentiated the herbivorous from the omnivorous strains, with an average error rate of 17%.

**Conclusions:**

This is the first work, as far as we are aware, that identifies the major source of fecal contamination of a pool of strains instead of a unique strain. We concluded that the analysis of the *E. coli *population structure can be useful as a supplementary bacterial source tracking tool.

## Background

*Escherichia coli*, a bacterium widely spread among warm-blooded animals, has been used as an indicator of water fecal contamination. Fecal pollution in water can indicate the presence of waterborne pathogens, such as *Salmonella *and *Giardia *[1]. The identification of the major animal source of fecal contamination is extremely important for the effective management of water systems [2]. Therefore, several methods of bacterial source tracking (BST), using *E. coli *strains, have been developed to identify the animal source of fecal contamination. Among these methods are ribotyping, rep-PCR, antibiotic resistance profiles, among others [3]. However, until now, only one putative human-specific strain [4] and one putative animal-specific strain have been found [5].

*Escherichia coli *strains can be assigned to one of the main phylogenetic groups: A, B1, B2 or D [6-8]. According to Lecointre *et al*. [9], groups A and B1 are sister groups whereas group B2 is included in an ancestral branch. These phylo-groups apparently differ in their ecological niches, life-history [10] and some characteristics, such as their ability to exploit different sugar sources, their antibiotic-resistance profiles and their growth rate [11]. Walk *et al*. [12] demonstrated that the majority of the *E. coli *strains that are able to persist in the environment belong to the B1 phylogenetic group. Furthermore, genome size differs among these phylo-groups, with A and B1 strains having smaller genomes than B2 or D strains [13]. Johnson *et al*. [14] found that strains from phylo-groups B2 and D contained more virulence factors than strains from the phylo-groups A and B1.

The extraintestinal pathogenic strains usually belong to groups B2 and D [15,16], the commensal strains to groups A and B1 [17], whilst the intestinal pathogenic strains belong to groups A, B1 and D [18]. Clermont *et al*. [19] have developed a PCR based method to characterize the phylo-groups using the genetic markers *chuA*, *yjaA *and the DNA fragment TspE4.C2. To increase the discrimination power of *E. coli *population analyses, it has been proposed the use of subgroups A_0_, A_1_, B1, B2_2_, B2_3_, D_1 _and D_2_, that are determined by the combination of the genetic markers [5].

Some authors analyzed the distribution of the main phylogenetic groups among *E. coli *strains isolated from human and animal feces. Gordon and Cowling [10] observed that the relative abundance of phylogenetic groups among mammals is dependent on the host diet, body mass and climate. Escobar-Páramo *et al*. [5] analyzing fecal strains isolated from birds, non-human mammals and humans, observed the prevalence of groups D and B1 in birds, A and B1 in non-human mammals, and A and B2 in humans. These authors concluded that one of the main forces that shapes the genetic structure of *E. coli *populations among the hosts is domestication. Baldy-Chudzik *et al*. [20] analyzed feces from zoo animals and found a prevalence of group B1 in herbivorous animals and a prevalence of group A in carnivorous and omnivorous animals.

The aim of this work was to analyze the distribution of phylogenetic groups and subgroups in feces from different animals and to assess the potential application of this analysis in identifying the major source of fecal contamination in the environment.

## Results

In this work, 241 *E. coli *strains isolated from feces of different animals and 12 strains isolated from a sewage source were allocated into four phylogenetic groups (i.e. A, B1, B2 and D) and seven subgroups (i.e. A_0_, A_1_, B1, B2_2_, B2_3_, D_1 _and D_2_). As shown in Table 1, the strains analyzed were distributed among the seven subgroups, and the prevalence indexes calculated for the subgroups were: A_0 _= 83.33%, A_1 _= 83.33%, B1 = 100%, B2_2 _= 50%, B2_3 _= 16.67%, D_1 _= 66.67 and D_2 _= 66.67%. It is interesting to note that strains from group B1 were found among all the analyzed hosts, whereas strains from subgroup B2_3 _were found only in humans.

**Table 1:** Distribution of the *E. coli *phylogenetic subgroups among the hosts analyzed

Phylogenetic subgroup	Human	Cow	Chicken	Pig	Sheep	Goat
A_0_	0	12	7	4	4	1
A_1_	38	2	3	17	0	2
B1	8	29	2	9	20	13
B2_2_	5	0	1	2	0	0
B2_3_	7	0	0	0	0	0
D_1_	26	4	0	5	3	0
D_2_	10	3	0	2	2	0
Total	94	50	13	39	29	16

**Figure 1:**
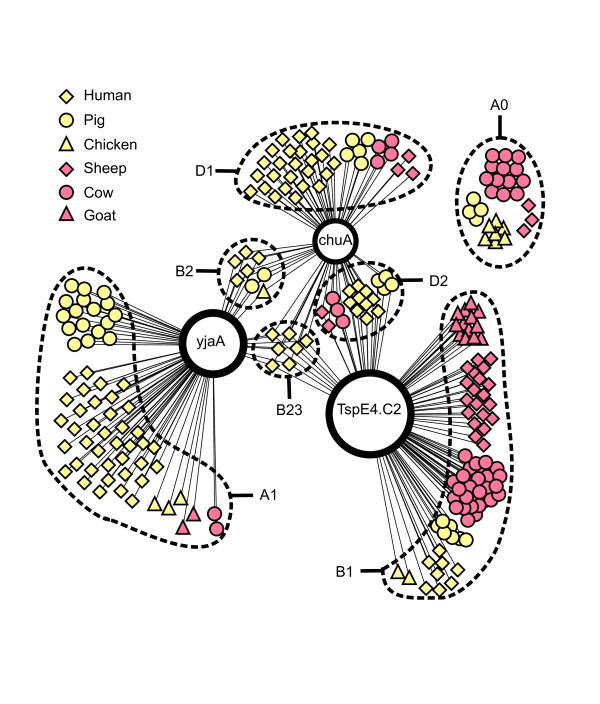
**Graphic representation of the occurrence of genetic markers in *E. coli *strains isolated from different hosts**. Large blank circles represent each genetic marker, *chuA*, *yjaA *and the DNA fragment TspE4.C2. Strains from different hosts are represented by different geometric shapes as described in the upper left. Strains from herbivorous animals are represented in pink and strains from omnivorous animals are in yellow. Edges between a strain and a genetic marker mean that the marker was detected for that strain. Each subgroup is highlighted by a dotted ellipse and labeled accordingly.

The graphic representation shown in Figure 1 allowed the identification of remarkable trends among the *E. coli *strains from the different hosts. Humans are the only host bearing strains from all the phylo-groups, except for subgroup A_0_. The strains found in the pig samples were also distributed among all phylo-groups, except for subgroup B2_3_, which contains only strains from the human samples. Most of the strains from the chicken samples were included in subgroup A_0_, that is, these strains did not reveal the presence of the genetic markers investigated. Most of the strains of cows, goats and sheep fell within group B1, despite the fact that four strains of cows and three of chickens were assigned to subgroup D_1 _and two strains of goats and two of cows were assigned to group A_1_.

A Chi-square value of 97.611, 15 degrees of freedom (D.F.), p < 0.0001, was obtained from a contingency table with the phylogenetic groups distribution among the hosts, allowing the null hypothesis, which states that there is no association between the hosts and the groups, to be rejected (p < 0.0001). This result suggests a significant difference in the *E. coli *population structure among the animals analyzed. A Chi-square test at the subgroup level was performed to verify the existence of an association between the hosts and the phylogenetic subgroup. The calculated 155.251 Chi-square value (30 D.F.), leads to the rejection of the null hypothesis (p < 0.0001). A Chi-square test was also performed to verify the association between the hosts and the genetic markers (*chuA*, *yjaA *and TspE4.C2). The result (Chi-square value = 87.563, 10 D.F., p < 0.0001) indicated that the genetic markers are differently distributed among the hosts (Table 2).

**Table 2:** Distribution of the *E. coli *genetic markers among the hosts analyzed

Genetic marker	Human	Cow	Chicken	Pig	Sheep	Goat	Total
*chuA*	48	7	1	9	5	0	70
*yjaA*	50	2	4	19	0	2	77
TspE4.C2	25	32	2	11	22	13	105

The Shannon and Simpson diversity indexes [21,22] were used to analyze the phylogenetic subgroup data. As shown in Table 3, the largest diversity indexes were observed for humans (Shannon index = 0.6598, Simpson index = 0.7331) and pigs (Shannon index = 0.6523, Simpson index = 0.7245), whilst the smallest diversity was observed for goats (Shannon index = 0.2614, Simpson index = 0.3203). The Pianka's similarity index was calculated using the phylogenetic subgroup distribution for each pair of hosts (Table 4). The results indicated that humans and pigs exhibited a similarity of 88.3%, whereas cows, goats and sheep exhibited an average similarity of 96%.

**Table 3:** Shannon's and Simpson's diversity index of each host analyzed

Diversity index	Human	Cow	Chicken	Pig	Sheep	Goat
Shannon index	0.6598	0.5029	0.5025	0.6523	0.412	0.2614
Simpson index	0.7331	0.5944	0.6272	0.7245	0.4899	0.3203

**Table 4:** Pairwise Pianka's index of similarity among the hosts analyzed

	Cow	Chicken	Pig	Sheep	Goat
**Human**	0.286	0.350	0.883	0.256	0.281
**Cow**	-	0.585	0.566	0.979	0.936
**Chicken**	-	-	0.609	0.414	0.372
**Pig**	-	-	-	0.507	0.574
**Sheep**	-	-	-	-	0.966

**Figure 2:**
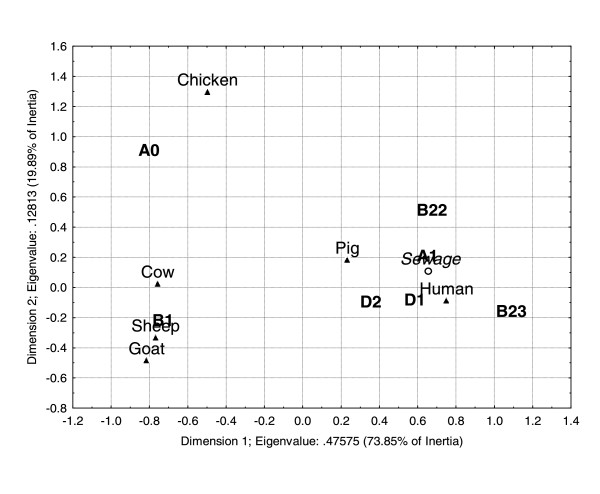
**Correspondence analysis using the contingence table of subgroup distribution among the hosts analyzed**. Subgroups and samples that are similar fall close. Eigenvalues are 0.47575 for the horizontal axis and 0.12813 for the vertical axis. The horizontal axis is responsible for 73.85% of the total inertia and the vertical axis for 19.89%.

A Correspondence Analysis (CA) was performed using the phylogenetic groups and subgroups distribution and the genetic markers distribution (Tables 1 and 2). The bidimensional representation of subgroups distribution in each host is shown in Figure 2. This bidimensional representation can explain 93.74% of the total inertia. The horizontal axis represents 73.85% of the total inertia, which is responsible for the major separation. According to this analysis, the subgroup distribution was similar for cows, goats and sheep and for pigs and humans (Figure 2). A sewage sample was included in the CA (Figure 2). This sample included the following subgroups: A_0 _(one strain), A_1 _(five strains), D_1 _(four strains) and D_2 _(two strains). As expected, this subgroup distribution was similar to the one found for humans (Figure 2).

**Figure 3:**
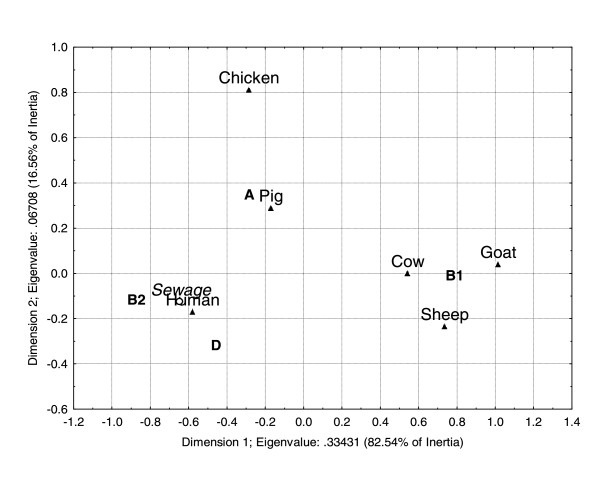
**Correspondence analysis using the contingence table of phylogenetic group distribution among the hosts analyzed**. Phylo-groups and samples that are similar fall close. Eigenvalues are 0.33431 for the horizontal axis and 0.06708 for the vertical axis. The horizontal axis is responsible for 82.54% of the total inertia and the vertical axis for 16.56%.

The CA using the genetic markers distribution resulted in a bidimensional representation that can explain 100% of the total inertia (Figure 3), being the horizontal axis responsible for 92.04% of it. According to this analysis, the genetic markers distribution was similar for cows, goats and sheep and for humans, chickens and pigs. The sewage sample, in which six strains presented the *chuA *gene, five the *yjaA *gene and two the TspE4.C2 fragment, was plotted near the human sample (Figure 3).

**Figure 4:**
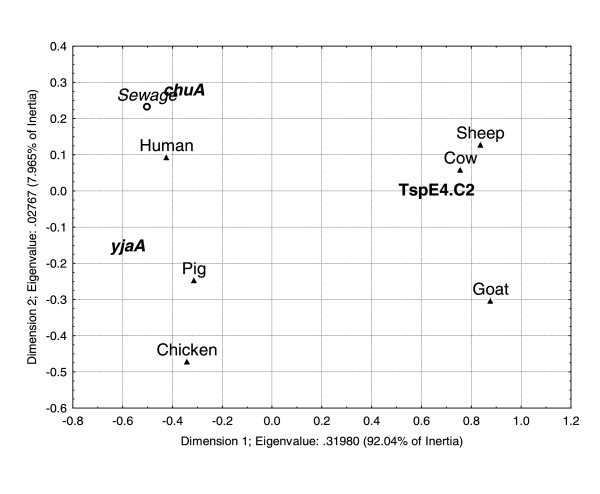
**Correspondence analysis using the contingence table of genetic markers distribution among the hosts analyzed**. Genetic markers and samples that are similar fall close. Eigenvalues are 0.31980 for the horizontal axis and 0.02767 for the vertical axis. The horizontal axis is responsible for 92.04% of the total inertia and the vertical axis for 7.965%.

The discrimination power of the phylogenetic groups A, B1, B2 and D was also tested using CA (Figure 4). According to this analysis, the bidimensional representation of the phylo-groups relative abundance can explain 99.1% of the total inertia, being the horizontal axis responsible for 82.54% of it. This analysis revealed that the phylo-group distribution among cows, goats and sheep, which presented a predominance of strains of the B1 group, was similar. Humans, chickens and pigs remained separated. *E. coli *strains isolated from two Rivers, Jaguari and Sorocaba, located in the State of São Paulo, Brazil, and previously analyzed by Orsi *et al*. [23], were also included in this CA analysis (data not shown). The strain composition of the Jaguari River included 42 strains of group A, 13 strains of group B1 and six strains of group D. The Sorocaba River included 45 strains of group A, 14 strains of group B1, one strain of group B2 and eight strains of group D. The strains distribution among the phylo-groups, from both rivers, was similar to the one observed for chickens and pigs. The sewage sample was also included in this CA and once again, this sample was similar to humans (Figure 4).

The results obtained with the classifier tools BLR and PLS-DA using the genetic markers are summarized in Table 5. The separation between *E. coli *strains of omnivorous and herbivorous mammals presented the lowest classification error rate (17% on average), while the highest classification error rate (25% on average) was observed between *E. coli *strains of humans and non-humans. Both classifier tools demonstrated that the *chuA *and the *yjaA *genes were more informative to discriminate between *E. coli *strains of human and non-human sources (data not shown). The PLS-DA tool showed that the *yjaA *gene and the TspE4.C2 DNA fragment were more informative to discriminate between *E. coli *strains of herbivorous and omnivorous mammals. The error rate for BLR and PLS-DA was higher in the prediction of human than in non-human samples (data not shown). However, when the feeding habit of mammals was considered in the separation, the error rate for both tools was higher in the prediction of the herbivorous samples.

**Table 5:** Classification error rates obtained by validation of supervised learning classifier tools (BLR and PLS-DA)

*E. coli *strains sources	Classifier tool	Overall cross-validation error rate	Overall test error rate
Humans and non-humans	BLR	22.50%	24.93%
	PLS-DA	25.33%	27.53%
Humans and non-humans mammals	BLR	22.09%	22.03%
	PLS-DA	22.09%	22.75%
Omnivorous and herbivorous mammals	BLR	16.57%	16.67%
	PLS-DA	18%	17.39%

The classification was carried out between human and animal samples, between humans and non-humans mammals and between omnivorous and herbivorous mammals

## Discussion and Conclusions

This study demonstrated that phylogenetic subgroup, group and genetic markers distribution are not randomly distributed among the hosts analyzed. The results showed a similarity between the *E. coli *population structure of humans and pigs (omnivorous mammals) and of cows, goats and sheep (herbivorous mammals). Humans and pigs exhibited the highest diversity indexes, while goats and sheep exhibited the lowest ones. Using the simulations of the EcoSim software [24], it was possible to conclude that the diversity indexes are significantly different among the herbivorous and omnivorous mammals. The Pianka's similarity index showed that the human sample was more similar to the pig sample (88.3% of overlap). Cows, goats and sheep also presented a high overlap (96% on average), while chickens presented the lowest values.

Cows, goats and sheep are ruminant mammals which differ in many gut characteristics from other animals. Humans and pigs present common gut characteristics because they are monogastric animals (reviewed in [25]). Besides the gut characteristics, the diet of the host appears to have selected the phylo-group profile in the Brazilian mammals analyzed in this work. Omnivorous mammals presented a prevalence of phylo-group A, while the herbivorous mammals presented a prevalence of phylo-group B1. Previous research by Gordon and Cowling [10] revealed a different result from ours, identifying a prevalence of strains of phylo-group B2 among herbivorous and omnivorous mammals and a prevalence of phylo-goup B1 among birds and carnivorous mammals, which supports their hypothesis of geographic effects in the *E. coli *population structure among hosts. However, they also concluded that phylo-groups A and B1 are "generalists" and B2 and D are "specialists", which is in agreement with our data since strains of group B1 were found in all the hosts analyzed, followed by subgroups A_0 _and A_1_. On the other hand, subgroup B2_3 _was present only in the human sample. Therefore, our results suggest that B2 strains, especially subgroup B2_3_, could be a good indicator of human feces contamination.

Group B1 was prevalent among the herbivorous hosts. However, this phylo-group is not a promising indicator of herbivorous feces contamination because it was found in all the hosts analyzed, and, apparently, most *E. coli *strains that are able to survive in the environment, belong to this group [12]. According to our data, the distribution analysis of phylo-groups A and D is a powerful discriminating tool since both groups presented a considerable contribution to the Chi-square values (data not shown).

The *chuA *and *yjaA *genes were rarely found in strains of cows, goats and sheep but were commonly found in human, chicken and pig strains. Sobieszczańska [26] showed that 95.5% of the enteroaggregative *E. coli *strains carried the *chuA *gene, which encodes for a haem receptor. Strains belonging to group B2 were not found in cows, goats and sheep. Other studies have demonstrated that B2 and D strains are usually more pathogenic than A and B1 strains [16,17,27,28]. In fact, verocytotoxin-producing *E. coli*, like O157:H7, belongs to group D [29] and cattle are the main reservoirs of this pathogen. The prevalence of groups B2 and D and of the *chuA *and *yjaA *genes in humans and pigs might suggest that fecal contamination by these animals can present a high risk of extra-intestinal pathogenic *E. coli*. Thus, the correct identification of this kind of fecal contamination can also be useful to the appropriate management of environmental pollution.

Correspondence analysis is a descriptive/exploratory technique, based on Chi-square values, that allows the exploration of the structure of the data. In the three CA models performed, similar distribution patterns were observed among *E. coli *strains of herbivorous mammals and among strains of omnivorous mammals. Furthermore, the CA of subgroup distribution allowed the discrimination of omnivorous mammals. Similar results were observed by Baldy-Chudzik *et al*. [20]. These authors suggested that the *E. coli *strains of group B1 are best adapted to herbivorous, whereas strains of group A are best adapted to omnivorous mammals. The three CA models correctly predicted the animal/human source of the external validation sample (sewage), indicating that a significant part of the *E. coli *phylo-group diversity was covered by the strains database, which reveals the stability of the models. *E. coli *samples from the Jaguari and Sorocaba Rivers [23] were also used to test the CA model based on phylo-group distribution. Our analysis suggested that pigs were the major source of fecal contamination in both rivers, which is in agreement with Orsi *et al*. [23], confirming that the major source of fecal contamination of these rivers was non-human. Therefore, these results indicate that the CA model can be efficiently applied in the discrimination of *E. coli *strains from different animal sources.

Both classifier tools (BLR and PLS-DA) and both validation methods (cross-validation and train-test) exhibited similar overall error rates for each strain separation analyzed. This way, the statistical method used did not show a significant interference in the obtained results. Excluding the chicken sample, the best classification was obtained when the *E. coli *strains were separated according to the feeding habits of the hosts (omnivorous and herbivorous mammals). Although the classification error rates found could be considered high, similar error rates were observed in other BST studies [30,31].

Since it is very difficult to find host-specific strains or genetic markers [4,32], in this work we propose a new approach to identify the animal source of fecal contamination in water systems. This approach is based on the specificity of the *E. coli *population structure instead of host-specific strains. Geographic variation of the *E. coli *population structure was reported in the literature [10,32] and since the relative abundance of phylo-groups among hosts can be easily characterized, this approach can be implemented in different regions of the world as a supplementary bacterial source tracking tool. Although our data is consistent in showing the potential applicability of this approach, we are aware that there might be some limitations due to the limited number of fecal pollution sources analyzed.

## Methods

The present study has been approved by the Research Ethics Committee of the State University of Campinas School of Medical Sciences.

### *Escherichia coli *Strains

Two hundred and forty one strains of *E. coli *were isolated (collected with sterile swabs) from fecal samples of a variety of hosts (Table 6). Each strain was isolated from a single animal. These strains were used to build the calibration set for further statistical analysis.

**Table 6:** Source and number of *E. coli *strains used in this study

Source	Number of Strains	References
Human	94	Gomes *et al*. [39]
Cow	50	Vicente *et al*. [40]
Chicken	13	Silveira *et al*. [41]
Pig	39	Isolated according to Vicente *et al*. [40]
Goat	16	Isolated according to Vicente *et al*. [40]
Sheep	29	Isolated according to Vicente *et al*. [40]
Sewage	12	Isolated by CETESB according to Orsi *et al*. [23]

Twelve sewage strains isolated by CETESB (Table 6), the organization responsible for the control of environmental pollution, sewage, and water quality in the State of São Paulo, Brazil, were used as the external validation set. The sewage samples were collected in 2008 at the Jesus Neto sewage treatment plant.

The strains were isolated as described by Orsi *et al*. [23], with modifications. Samples were analyzed using the membrane filter technique with modified mTEC agar (Difco) and incubated for 2 h at 35 ± 0.5°C and 22--24 h at 44.5 ± 0.2°C. Typical colonies were streaked on EMB agar (Merck). Isolated colonies were tested for citrate utilization, lactose fermentation, oxidase, l-lysine decarboxylase, motility, glucose and sucrose fermentation, tryptophan deamination, indole production, urea hydrolysis and sulfide production. Isolates with an *E. coli *profile were inoculated into LB broth at 37°C overnight. One isolated colony from each EC positive sample was selected for further analyses.

### Phylogenetic group determination

The phylogenetic group of each strain was determined according to Clermont *et al*. [19], by multiplex PCR of the genes *chuA *and *yjaA *and the DNA fragment TspE4.C2. The amplification products were separated in 2% agarose gels containing ethidium bromide [33]. After electrophoresis, the gel was photographed under UV light, and the strains were assigned to the phylogenetic groups B2 (*chuA*+, *yjaA*+), D (*chuA*+, *yjaA*-), B1 (*chuA*-, TspE4.C2+) or A (*chuA*-, TspE4.C2-).

To increase the strains discrimination, subgroups or phylotypes were determined as follows: subgroup A_0 _(group A), *chu*A-, *yja*A-, TspE4.C2-; subgroup A_1 _(group A), *chu*A-, *yj*aA+ TspE4.C2-; group B1, *chu*A-, *yja*A-, TspE4.C2+; subgroup B2_2 _(group B2), *chu*A+, *yja*A+, TspE4.C2-; subgroup B2_3 _(group B2), *chu*A+, *yja*A+, TspE4.C2+; subgroup D_1 _(group D), *chu*A+, *yja*A-, TspE4.C2- and subgroup D_2 _(group D), *chu*A+, yjA-, TspE4.C2+ [5].

### Bioinformatic and statistical analysis

A graphic representation was used to map the occurrence of the genetic markers *chuA*, *yjaA *and TspE4.C2 in the *E. coli *strains isolated from the different hosts. For this, the genetic markers were scored as present/absent in each strain analyzed, and the graphic was drawn with the software Pajek v. 1.22 http://vlado.fmf.uni-lj.si/pub/networks/pajek/. This graphic provides a useful representation of the *E. coli *phylo-groups among the different hosts. It contains two sets of nodes -- genetic markers and samples -- and edges between them. An edge between two nodes means that the genetic marker was detected for a given strain.

The prevalence index (P) was calculated by dividing the number of hosts exhibiting a particular subgroup by the total number of hosts analyzed. The results were expressed as percentages [34].

The Pianka's index was calculated to evaluate the subgroup overlap between two hosts by using the formula: O = ∑*p_j_p_k_*/√∑*p_j_*^2^∑*p_k_*^2^, where *pj *and *pk *are subgroups proportions in the hosts *j *and *k*, respectively. The results were expressed as percentages [35].

The Chi-square test, the Simpson's diversity index and the Shannon's index were performed with the BioEstat v. 5.0 software [36], using the phylogenetic subgroup data. The EcoSim software [24] was used to test the differences among the diversity indexes by using resampling. The frequencies of phylogenetic groups, subgroups and genetic markers were compared among the hosts by using the CA, which was performed by using STATISTICA 6.0 [37]. The sewage sample was used to challenge the CA models as an external validation sample.

The classifier tools Binary Logistic Regression (BLR) and Partial Least Saquares -- Discriminant Analysis (PLS-DA) were performed with the software TANAGRA 1.4 [38]. For these analyses, the hosts were separated into humans and non-humans, human and non-human mammals, omnivorous and herbivorous mammals. The genetic markers were scored as present/absent. The cross-validation of these analyses was carried out by using five repetitions and ten fold parameters, and the train-test was carried out using 70% of the samples as a training set and ten repetitions of assessment.

## Authors' contributions

CC and LMMO conceived and designed the study. CC performed the experiments, the statistical analysis and wrote the manuscript. MMP performed the bioinformatic analysis. NCS, TATG and LAA isolated the majority of the *E. coli *strains used in the work. MIZS and EMH participated in the discussion of the experimental results. All authors read and approved the final manuscript.
